# Work-related musculoskeletal disorders and contributing factors among social security workers in Dar es Salaam: a cross-sectional study

**DOI:** 10.3389/fpubh.2026.1852675

**Published:** 2026-06-05

**Authors:** Simon Lwaho, Edson Protas, Evaline D. Asenga, Suleiman Chombo, Peter M. Chilipweli, Luco Mwelange, Israel Nyarubeli

**Affiliations:** 1 Directorate of Training, Research, Statistics and Promotion, Occupational Safety and Health Authority, Dar es Salaam, Tanzania; 2 Department of Environmental and Occupational Health, Muhimbili University of Health and Allied Sciences, Dar es Salaam, Tanzania; 3 Department of Occupational Health (Port Health), Julius Nyerere International Airport, Dar es Salaam, Tanzania; 4 Department of Community Medicine and Environmental Occupational Health, Catholic University of Health and Allied Sciences, Mwanza, Tanzania

**Keywords:** Dar es Salaam, ergonomics, office work, social security workers, work related musculoskeletal disorders (MSDs)

## Abstract

**Background:**

Work-related musculoskeletal disorders (MSDs) are a significant public health problem for individuals engaged in tasks involving repetitive upper limb movements and extended computer use, including those working in the social security sector. The International Labor Organization (ILO) has listed musculoskeletal disorders as an occupational disease that requires preventive measures in workplaces. However, there is limited information in the context of Tanzania. Therefore, this study aimed to address this knowledge gap, focusing on the magnitude and risk factors of MSDs among social security workers in Dar es Salaam.

**Objective:**

This study aimed to assess the work-related musculoskeletal disorders and the contributing risk factors among social security workers in Dar es Salaam.

**Materials and methods:**

The study used a cross-sectional design and included a sample of 300 employed social security workers working at NSSF offices in the Dar es Salaam region. The study included all NSSF workplaces in Dar es Salaam, and eligible participants were selected using probability proportional to size. Data were collected using a structured Nordic questionnaire to determine the prevalence and factors contributing to MSDs. In contrast, a checklist was used to identify preventive measures and factors contributing to MSDs. A modified Poisson model of analysis was used to analyse the factors contributing to MSDs.

**Results:**

The overall prevalence of MSDs reported by participants in the previous 12 months was 77.7%. The significant risk factors for MSDs included lower education levels (aRR: 1.71; 95% CI: 1.140–2.564, *p* ≤ 0.010), job categories (aRR: 0.65; 95% CI: 0.452–0.939, *p* = 0.022) in administrative sectors and (aRR: 0.68; 95% CI: 0. 0.486–0.952, *p* = 0.025) compliance sectors, level of activity (aRR: 0.337; 95% CI: 0.200–0.571, *p* < 0.001), repetitive tasks (aRR: 1.5; 95% CI: 1.197–1.912, *p* = 0.001), awkward posture (aRR: 0.634; 95% CI: 0.502–0.801, *p* = 0.010), and lack of ergonomic training (aRR: 1.46; 95% CI: 1.108–1.918, *p* = 0.007). Furthermore, more than three-quarters of social security workers used chairs with adjustable height and back support and had working surfaces with adequate space. However, less than half of these workers had not received the ergonomic training.

**Conclusion and recommendations:**

The results of this study reveal a high prevalence of MSDs among social security workers in Dar es Salaam. These findings serve as a crucial alert for employers and employees to develop and review existing ergonomic interventions. It is also recommended that the government strengthen and regularly conduct ergonomic training, inspections, and enforcement in all workplaces.

## Introduction

Musculoskeletal disorders (MSDs) are conditions, either acute or chronic, that affect the body’s muscles, tendons, nerves, and supporting structures, leading to impaired function (1). The etiology of these disorders is complex and can partly be attributed to exposure to various ergonomic hazards and environmental factors (2). The International Labor Organization (ILO) has classified work-related musculoskeletal disorders as an occupational disease that requires preventive measures in workplaces (3).

Social security has been widely adopted by several developing countries, including Tanzania, as a means to improve living standards and promote economic growth through saving mobilization and income redistribution (4). Recently, Tanzania has reformed its social security sector, establishing two major schemes: the Public Service Social Security Fund (PSSSF) for serving public servants and the National Social Security Fund (NSSF) for serving private-sector employees (5). The primary functions and core operations of workers involved in these schemes include registering members, collecting contributions, investing the collected funds, and paying benefits to members. With technological advancements, the majority of these roles have been digitalized and now primarily consist of office-based tasks, with the exception of inspection roles that require field visits sometimes (6).

Musculoskeletal disorders (MSDs) are a frequently reported issue in various workplace settings, particularly among individuals engaged in stationary tasks or activities that involve repetitive upper limb movements and extended periods of computer use (7–10). Studies conducted in Ireland, Iran, Kuwait, India, and Ethiopia have reported the vulnerability of several office worker populations to MSDs. Office workers are particularly susceptible to work-related musculoskeletal disorders due to prolonged sitting, extensive computer use, and repetitive tasks (11). A study involving bank workers in Kuwait found that office work settings and activities contribute to several factors leading to musculoskeletal disorders and increased absenteeism (12). Evidence suggests that personal behaviors, such as body mass index (BMI), low job control, awkward postures, repetitive tasks, lack of training, and long job tenure, may play a significant role in the development of work-related musculoskeletal disorders among office workers, thereby increasing the rates of sick leave among employees.

In sub-Saharan countries and Tanzania, few studies have been conducted in assessing MSDs to other professionals, excluding social security workers who are part of an office working group with heavy interaction in desk works (13–15). Furthermore, previous research efforts of studying the Occupational Health and Safety (OHS) status in Tanzania have recommended studies to be conducted in this area so as to generate evidence-based information which can help provide solutions to the majority of OHS problems (16). Therefore, this study focuses on establishing the prevalence of work-related musculoskeletal disorders and the contributing factors to office workers’ populations, particularly social security workers based in the Dar es Salaam region.

## Methodology

### Study design and population

A cross-sectional study design was used, with the aim of collecting both work-related musculoskeletal disorders and the contributing factors among social security workers. The study was conducted in the Dar es Salaam region, which was established by the colonial government as a township since 1920 and has remained an important commercial city and a business hub within Tanzania and neighboring countries. According to the 2022 population census, the city had a population of 5,383,728. The integrated labor force survey conducted in Tanzania 2020/21 revealed Dar es Salaam as the leading region with a proportion of 76.4% of the active population engaged in the labor market (17). Dar es Salaam has a great representative ability among other regions in this study because it comprises a large number of the employed social security workers as compared to other regions (17). Apart from other office workers, social security workers were selected for this study due to the high prevalence of computer use, as most of the operations have been digitalized (6, 17). This population comprises a diverse group of professionals engaged in various administrative, clerical, and office-based roles within the social security sector. Furthermore, the study included office-based workers who had been working for at least 1 year (12 months) in social security operations and excluded workers with a history of any accidents that resulted in muscular injuries and fractures, as well as workers with disability and pregnant women.

### Sample size and sampling

The sample size for this study was calculated by using the Cochran formula (18), with a known population of 831, the total number of employees of the social security in the Dar es Salaam region. The formula included a margin of error (5%), which allowed for a reasonable level of precision in the estimated sample size. The formula also accounted for potential non-response by factoring in a 10% non-response rate. Therefore, the final sample size included for this study was 300 social security workers who are based in the Dar es Salaam region.

The study participants were selected from all seven workplaces (six branches and one head office) of the social security office, which are located in every council of the Dar es Salaam region. The number of study participants selected from each workplace was determined using probability proportional to size. All eligible participants present on the data collection day were sampled until the required sample size was achieved for the respective workplace. The data collection was conducted for the period of 4 months from March to July 2024.

### Data collection tool

A study utilized a structured questionnaire categorized into three parts: social-demographic data, and the second part of the questionnaire included factors that contribute to the development of work-related MSDs. The third part of the questionnaire included the Nordic technique of assessing the musculoskeletal discomforts, which categorized the human body into nine regions, prompting study participants to report discomfort in the respective body region (19).

### Data management and analysis

The study engaged three trained research assistants responsible for data collection, quality assurance, and management. Data were entered and coded using SPSS software. Data cleaning and verification were conducted through frequency tables to assess completeness and consistency. Questionnaires with missing data were excluded from the analysis. Statistical analysis was performed using SPSS.

The dependent variable was the presence of musculoskeletal disorders, including pain, discomfort, impairment, and disability. This outcome was assessed using a binary (yes/no) response to whether any pain, ache, or discomfort was reported in one or more of nine predefined body regions within the past 12 months (19–21).

Independent variables included sex, age, job category, physical exercise, level of activity, repetitive tasks, hours worked per week, duration of employment, awkward postures, availability of facilities, work breaks, presence of a chair with adjustable height and back support, space under the table, and use of a round-edged table.

Descriptive statistics were used, including frequency distributions, to determine the proportion of work-related musculoskeletal disorders (MSDs). Pearson’s chi-square test was used to assess the association between the presence of MSDs (yes/no) and the categorical explanatory variable at a 5% significance level.

To evaluate the relationship between contributing factors and the outcome (presence of any reported MSDs), a modified Poisson regression model with robust standard errors was used. Initially, univariable modified Poisson regression was conducted, and variables with a -value less than 0.2 were included in the final multivariable modified Poisson regression model to adjust for potential confounders. Variables with value less than 0.05 at a 95% confidence interval were considered statistically significant.

### Ethical issues

The ethical clearance was applied to the MUHAS Institutional Review Board (IRB) permit no. MUHAS-REC-03-2024-2094 before the data collection, while the permission to collect data was granted by the Director General of NSSF through a letter with reference number CA.259/314/01/69. The study participants who took part in the study were given clearly awareness on the main purpose of the study and given formal consent to willingly approve their participation. Furthermore, the information collected from workers were maintained in high confidentiality.

## Results

### Socio-demographic characteristics of respondents

A total of 300 social security workers from the Dar es Salaam region participated in the study, achieving a 100% response rate. The sample displayed an almost equal distribution across genders, with males (49%, *n* = 147). The majority of respondents had an age ranging between 30 and 39 years (57%, *n* = 172). Based on educational levels, a significant number of the respondents had a bachelor’s degree (75%, *n* = 226). In terms of physical exercise, 36% of the study participants (*n* = 107) engaged in weekly physical activity. The job category distribution showed that half of the study participants reported having worked in compliance-related roles (Table 1).

**Table 1:** Socio-demographic characteristics of respondents (*n* = 300).

Variable	Category	*n* (%)
Sex	Male	147 (49.0)
	Female	153 (51.0)
Age group	20–29	65 (21.7)
	30–39	172 (57.3)
	40 and above	63 (21.0)
Education level	Certificate	32 (10.7)
	Diploma	42 (14.0)
	Any degree	226 (75.3)
Physical exercise (lifestyle)	Daily	61 (20.3)
	Weekly	107 (35.7)
	Monthly	57 (19.0)
	Not doing at all	75 (25.0)
Job category	Benefit processing	30 (10.0)
	Compliance	150 (50.0)
	Administrations	45 (15.0)
	Other*	75 (25.0)

Other*, included other reported job categories such as drivers, doctors, engineers, and estate officers.

### Magnitude of the work-related musculoskeletal disorders among social security workers

**Figure 1:**
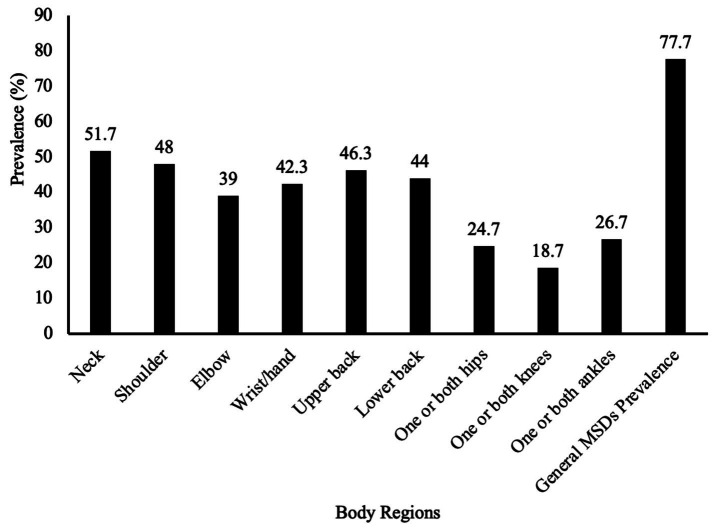
Prevalence (%) of reported body regions affected by MSDs among social security workers in Dar es Salaam, 2024.

Overall, the proportion of any reported MSDs from the study participants for the last 12 months was 78% (*n* = 233). When considering the upper extremities, 52% (*n* = 155) reported neck pain, and 48% (*n* = 144) reported shoulder pain. This was closely followed by the upper back pain 46% (*n* = 139) and lower back pain at 44% (*n* = 132). The prevalence of wrist/hand pain was slightly higher at 42% (*n* = 127) compared to the elbow pain 39% (*n* = 117). Pain in the lower extremities was reported to a lesser extent, as detailed in Figure 1.

### Contributing factors to musculoskeletal disorders among the social security workers

The modified Poisson regression model identified 13 out of 15 factors as significantly associated with musculoskeletal disorders at a *p*-value of <0.2. The significant factors were included at a higher level in the multivariable modified Poisson analysis model to adjust for confounders.

The results from the multivariable modified Poisson regression analysis indicated that participants with a diploma level had 71% higher risks of having any form of musculoskeletal disorders as compared to participants with a certificate level (95% CI: 1.140–2.564, *p* ≤ 0.010). Participants engaging in administrative and compliance works had 35% (95% CI, 0.452–0.939, *p* = 0.022) and 32% (95% CI, 0. 0.486–0.952, *p* = 0.025) less risk of having any form of musculoskeletal disorders compared to participants engaged in benefit processing work, respectively.

The level of activity category has found that the field workers’ category had a significant reduction in musculoskeletal disorders, with a lower risk of aRR 0.337 (95% CI: 0.200–0.571, *p* < 0.001) compared to full office workers. Daily work that includes repetitive tasks had a significant increase in MSDs with aRR of 1.5 (95% CI: 1.197–1.912, *p* = 0.001). Study participants who commonly sit in awkward postures doing tasks that involve body twisting have a higher risk, in contrast to workers who do not twist their bodies, wo have a significant risk of musculoskeletal disorders, with aRR of 0.634 (95% CI: 0.502–0.801, *p* = 0.010), and finally, lack of ergonomic training was significant to MSDs by aRR of 1.46 (95% CI: 1.108–1.918, *p* = 0.007) compared to participants who had received the training concerning ergonomics (Table 2).

**Table 2:** Factors contributing to musculoskeletal disorders among social security workers at Dar es Salaam (*n* = 300).

Variable	Category	Crude RR (95%CI)	*p*-value	Adjusted RR (95%CI)	*p*-value
Sex	Male	1		1	
	Female	1.06 (0.94–1.19)	0.382	0.912 (0.711–1.170)	0.471
Age group	20–29	1			
	30–39	1.12 (0.94–1.33)	0.213	1.026 (0.827–1.273)	0.814
	40 and above	1.14 (0.94–1.40)	0.181	1.049 (0.777–1.417)	0.754
Education level	Certificate	1			
	Diploma	0.93 (0.85–1.01)	0.084	1.71 (1.140–2.564)	0.010***
	Any degree	0.72 (0.66–0.78)	<0.001	0.71 (0.497–1.028)	0.070
Job category	Benefit processing	1			
	Compliance	0.75 (0.69–0.82)	<0.001	0.68 (0.486–0.952)	0.025***
	Administrations	0.67 (0.54-0.82)	<0.001	0.65 (0.452–0.939)	0.022***
	Other**	0.80 (0.71-0.90)	<0.001	0.82 (0.568–1.191)	0.302
Physical exercise	Daily	1		1	
	Weekly	1.18 (0.98–1.42)	0.077	0.772 (0.510–1.168)	0.220
	Monthly	1.05 (0.84–1.31)	0.700	0.783 (0.521–1.179)	0.242
	Not doing at all	1.15 (0.91–1.36)	0.285	1.184 (0.889–1.577)	0.248
Level of activity	Full office	1		1	
	Office and field	0.80 (0.69–0.92)	0.002	0.593 (0.438–0.803)	0.001***
	Field	0.74 (0.57-0.96)	0.026	0.337 (0.200–0.571)	<0.001***
Repetitive works	Hourly	1		1	
	Daily	1.14 (0.97–1.34)	0.102	1.513 (1.197–1.912)	0.001***
	Weekly	1.45 (1.27- 1.67)	<0.001	1.184 (0.796–1.762)	0.405
	Monthly	1.21 (0.95–1.55)	0.131	0.839 (0.493–1.428)	0.518
Employment duration	1 year and below	1			
	Above 1 year	0.97 (0.75–1.26)	0.815		
Working hours/week	Below 40 h	1			
	Above 40 h	1.07 (0.94–1.23)	0.289		
Ergonomic training	Received	1		1	
	Not received	1.37 (1.14–1.66)	0.001	1.46 (1.108–1.918)	0.007***
Awkward posture	Yes	1			
	No	0.74 (0.64–0.86)	<0.001	0.634 (0.502–0.801)	<0.001***
Chair with adjustable height and back	Yes	1		1	
	No	1.31 (1.18–1.46)	<0.001	0.867 (0.686–1.095)	0.232
Space under the table	Adequate	1		1	
	Inadequate	1.27 (1.14–1.42)	<0.001	0.966 (0.748–1.247)	0.792

*** Significant factors *p* < 0.05.

## Discussion

The overall prevalence of MSDs over the last 12 months was found to be 77.7%. These results are consistent with studies conducted in other developing countries, for instance, a study in Addis Ababa among bank workers reported a prevalence of 77.6% (21). Similarly, bank workers in Kuwait and Iranian office workers had reported an increased prevalence of MSDs, that is, 83 and 88%, respectively (22, 23). However, a few studies have reported a lower prevalence, nearly or below 50% of study participants (20). The marked difference in prevalence might be attributed to various factors such as social and cultural aspects, technological levels, work targets, methods of assessments, and the nature of activity done by participants. For the Tanzania case, the reported prevalence might be influenced by limited ergonomic facilities, low level of technology, limited awareness among workers and employers, as well as inadequate workplace ergonomic survey and surveillance systems.

This study has also determined the body regions’ prevalence among social security workers, the four most common body regions with high prevalence of musculoskeletal disorders were neck pains, shoulder pains, upper back pain, and low back pain. This finding is similar to several studies that have been conducted worldwide (2, 12, 20, 22, 24, 25). This pattern is consistent with the physical demands of office work, where prolonged sitting and computer use can strain these regions, as observed in a study by (22). The hips, knees, and ankles were less reported compared to the upper body regions. This could indicate that there is a low impact on the lower extremities, and this is similar to the results obtained in the study conducted in India (20). Generally, these findings imply that more than half of the workers’ population in office settings suffer from this condition, and this comparison highlights the significant ergonomic and occupational health challenges faced by social security workers, therefore necessitating the establishment of ergonomic and wellness programs to protect the health of social security workers and reduce the burden due to increased prevalence of MSDs among social security workers.

Education level has been found to be a significant predictive factor of musculoskeletal disorders, where category analysis shows that the increase in education level attainment decreases the risk of musculoskeletal disorders. This study has determined the lower risk to participants with any degree level education compared to those with certificate education. This finding is similar to the study conducted among bank workers in Ethiopia, which reported that low-level educated participants had a higher risk of developing work-related musculoskeletal disorders as compared to highly educated bank workers (2). This finding might imply that most workers with low education status are probably exposed to ergonomic hazardous activities in the workplace, such as tasks that are physically demanding, prolonged sitting, manual handling of objects, working in awkward postures, and strenuous postures. However, several studies did not find the significance of education level. This might be due to differences in socio-cultural characteristics, workplace settings, and development in technologies that simplify work to be done in an ergonomic-friendly way (20, 21).

Job category analysis was significantly associated with the likelihood of work-related musculoskeletal disorders; the administration and compliance work sections had a lower risk of musculoskeletal disorders compared to the benefit processing work sections. These results are consistent with other global studies (10). The protective effect in administrative roles might be due to more controlled environments and better ergonomic facilities layout in front offices, while in benefits processing sections, they are busy with operational work that involves physical strains, prolonged sitting, improper layouts, and housekeeping. The workers who are engaged in compliance job roles have the protective factor of avoiding prolonged office work by combining office work and field work routinely. Therefore, this raises the concern for the management of social security funds to improve the work organization and workplace layout to meet ergonomic requirements.

The current study has found that the level of activity in the work schedule was a significant factor for MSDs, whereby workers in the categories that are engaged in office and field work, and the category of workers who are doing field work only, had a lower risk compared to workers who were engaged fully in office work. This finding highlights that task diversifying can be a protective factor against musculoskeletal disorders as it will help to improve muscle balance and decrease body fatigue. These results emphasize the role of job rotation and task variation in reducing the incidence of musculoskeletal disorders among social security workers. The global studies suggest that job rotations promote better muscle conditioning and prevent the risks of musculoskeletal disorders (26, 27).

In this study, repetitive work was found to have an increased risk for MSDs. These findings are supported by the recent study that has mentioned the repetitive work as a significant factor of work-related musculoskeletal disorders (28). This finding also underscores the role of intensity and frequency in developing musculoskeletal disorders, especially among the highly exposed social security workers who are engaged in activities such as routine typing. Therefore, the emphasis shall be put on proper job designing and automation of work processes to minimize the daily exposure to repetitive work of social security workers.

This study of social security workers in Dar es Salaam has also found that workers who work in a neutral bodily posture have a lower risk of musculoskeletal disorders compared to workers who twist their bodies while working at their work stations. A study conducted in Ethiopia reported that the high prevalence of neck and shoulder disorders was related to the posture maintained during computer use, which often involves forward head posture and raised shoulders. Upper and lower back issues may arise from prolonged sitting and body twisting (21). A study conducted among bank workers in Ethiopia has found that those with bad working conditions have a higher risk of developing work-related musculoskeletal disorders. This comparison of findings shows that the awkward sitting posture that involves body twisting is a major factor contributing to MSDs among office workers. However, maintaining correct posture during prolonged computer use is challenging, leading to habits such as slouching and leg crossing among most office workers. If incorrect postures become a habit at an early age, individuals maintaining those postures may adapt and consider them comfortable, and this can cause strain on the spine, pelvis, muscles, tendons, joints, bones, and discs, which can lead to fatigue, deformation, and dysfunction (22). Thus, incorrect habits, such as excessive body twisting while using computers, improper adjustment of desks and chairs to the proper height, and any form of inappropriate postures, affect the shape of muscles, deform the skeleton, and cause abnormal development, which prohibit the maintenance of correct posture (11). To prevent these health problems, the sitting behavior of office workers must undergo critical ergonomic interventions to prevent MSDs among social security workers.

This study has found that ergonomic training was a significant factor toward musculoskeletal disorders (MSDs) among social security workers in Dar es Salaam. Workers who did not receive ergonomic training had a higher risk of MSDs compared to workers who received ergonomic training. This finding aligns with a study conducted in Ethiopia that revealed a higher risk of developing musculoskeletal disorders among bank workers who did not have ergonomic training (2). This comparison implies the role of ergonomic training as an important aspect of the ergonomic intervention programs in the workplace. Furthermore, this can be supported by other findings of this study, whereby only less than half of the social security workers who participated in the study have received ergonomic training, which might be the potential reason why the prevalence of musculoskeletal disorders among social security workers is still more than three-quarters. This finding underscores the critical importance of ergonomic awareness and training in protecting workers against musculoskeletal disorders.

### Strength and limitation

The current study had strengths such as a sufficient participation rate of 100% of social security workers in Dar es Salaam, a high participation rate, which eliminated the risk of non-response bias and enhanced the validity of the study findings. Moreover, the study utilized the validated Nordic musculoskeletal questionnaire (NMQ), which is a widely recognized tool for assessing musculoskeletal disorders. Using this tool in a study offers confidence that the collected data are reliable and comparable with other studies that used the same tool, further strengthening the credibility of the results. However, this study was not free from limitations, such as possible symptom recall bias at 12 months and the cross-sectional design of the study, which restricts the ability to establish causal relationships between risk factors and musculoskeletal disorders. This design only provides a snapshot in time, making it difficult to determine whether the identified risk factors preceded the development of musculoskeletal disorders or vice versa.

### Conclusion and recommendation

The findings of this study have shown that social security workers in Dar es Salaam had reported high prevalence of work-related musculoskeletal disorders (MSDs) in at least one region of the body over the past 12 months. The most commonly affected body regions were the neck, shoulder, and lower back. Multivariate analysis of the modified Poisson model indicated that significant predictors for the occurrence of MSDs among these workers included educational level, job category in their workplaces, full office-based activity, repetitive work performed daily, lack of ergonomic training, as well as awkward posture in work that involves body twisting.

To reduce the prevalence of MSDs, it is recommended that social security offices implement proper ergonomic interventions, including the use of adjustable chairs for all workers with prolonged sitting tasks, workers to maintain appropriate work postures, regular breaks, and comprehensive wellness programs. Awareness and training programs on maintaining healthy working conditions and ergonomic practices are also crucial to prevent MSDs. Furthermore, social security workers shall arrange their work in such a way that they avoid tasks that require body twisting and train their bodies to adapt to good working postures when seated or standing during routine activities in their work sections. The government shall strengthen and conduct ergonomic training for all workers, emphasize regular ergonomic inspection and enforcement in all workplaces, and ensure that the implementation of the preventive intervention in the social security sector is adequate to protect the health, safety, and wellbeing of the workers.

## Data Availability

The raw data supporting the conclusions of this article will be made available by the authors, without undue reservation.
